# Supplementary material for: Forgive, Let Go, and Stay Well! The Relationship between Forgiveness and Physical and Mental Health in Women and Men: The Mediating Role of Self-Consciousness
Source: Int J Environ Res Public Health. 2023 Jun 26;20(13):6229. doi: 10.3390/ijerph20136229 (PMC10341467; doi:10.3390/ijerph20136229)
Supplement: Supplementary file 1 [file ijerph-20-06229-s001.zip › ijerph-2360613-supplementary.pdf]

Supplementary Materials

Forgiveness on dimension of general health via rumination and reflection

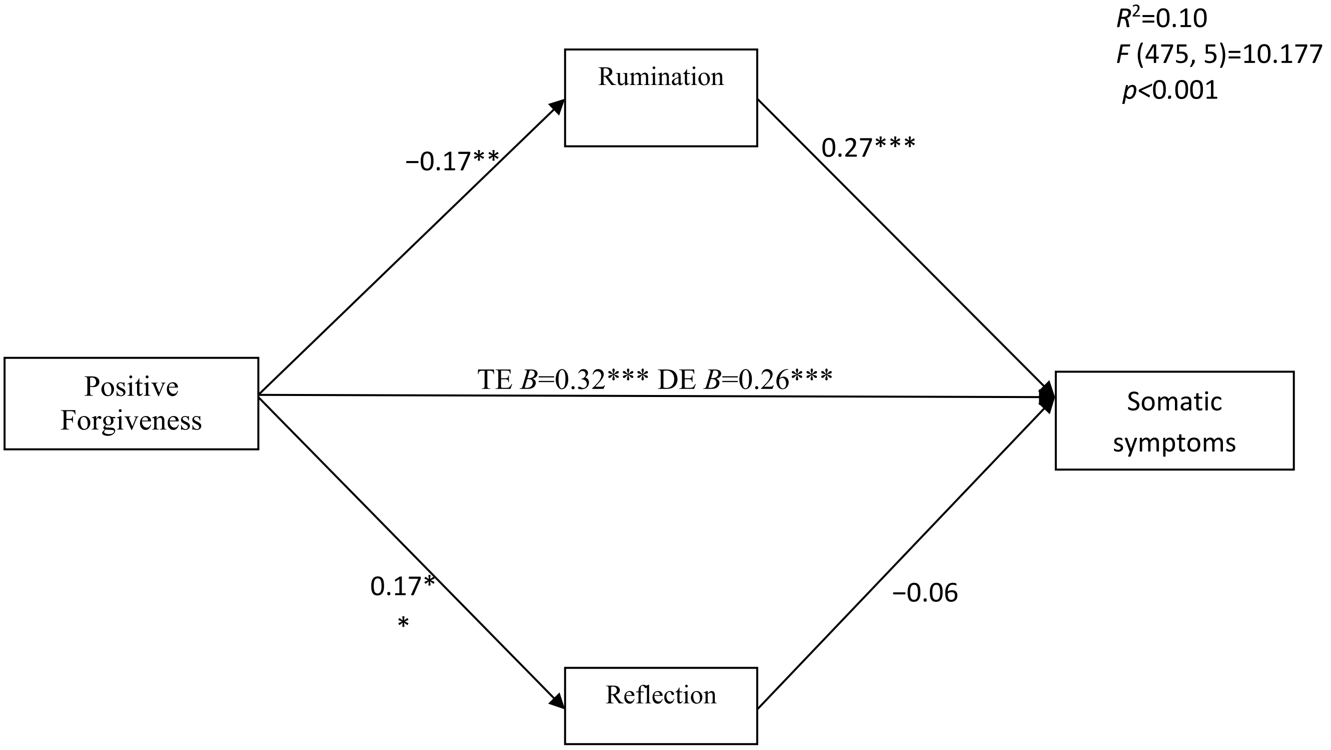

Figure S1 The indirect effect of Positive forgiveness on somatic symptoms rumination and reflection .

*$p<0.05$; **$p<0.01$; ***$p<0.001$

Indirect Effect (IE) (via Rumination and Reflection) – $B=-0.056$ $CI_{95\%}$[−0.093, −0.024]

IE (via Rumination) – $B=-0.047$ $CI_{95\%}$[−0.079, −0.021]

IE (via Reflection) – $B=-0.009$ $CI_{95\%}$[−0.028, 0.005]

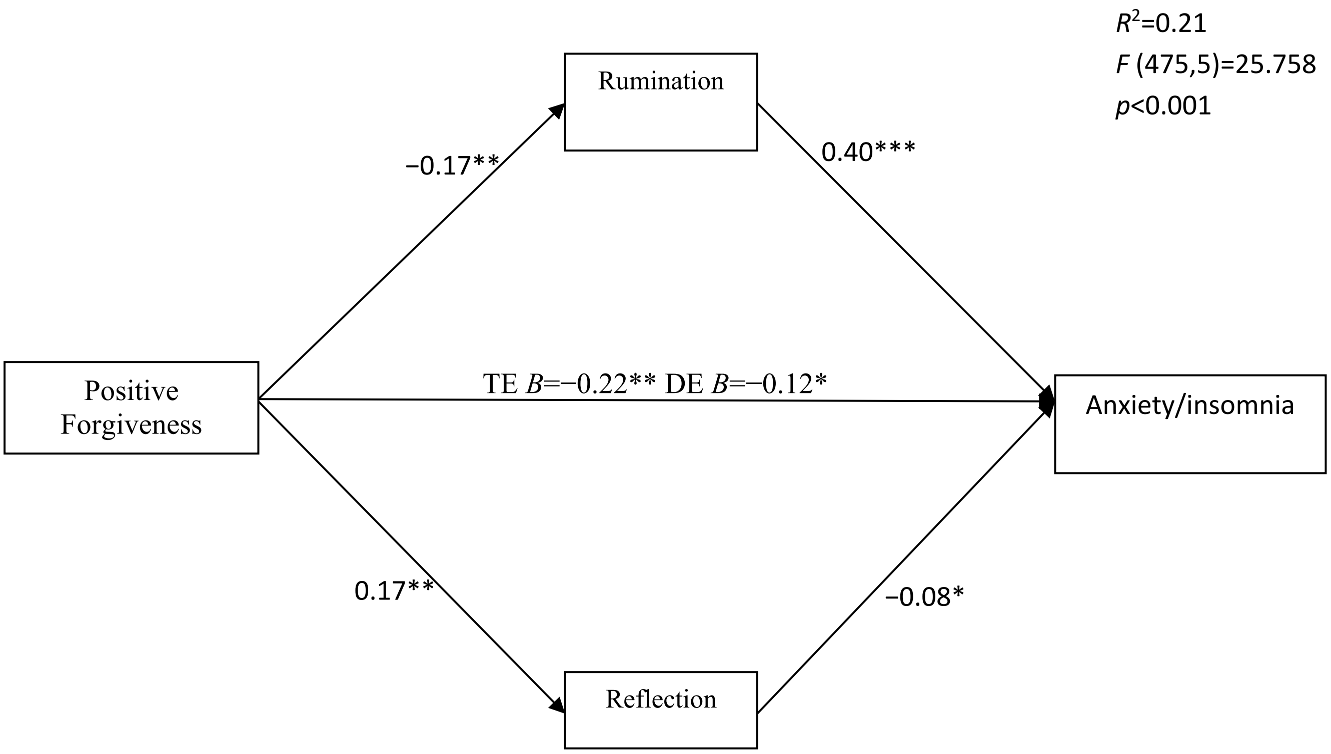

Figure. S2 The indirect effect of positive forgiveness on anxiety/insomnia via rumination and reflection.

*p<0.05; **p<0.01; ***p<0.001

IE (via Rumination and Reflection) – B=−0.083 $CI_{95\%}$[−0.123, −0.046]

IE (via Rumination) – B=−0.068 $CI_{95\%}$[−0.105, −0.034]

IE (via Reflection) – B=−0.015 $CI_{95\%}$[−0.035, −0.001]

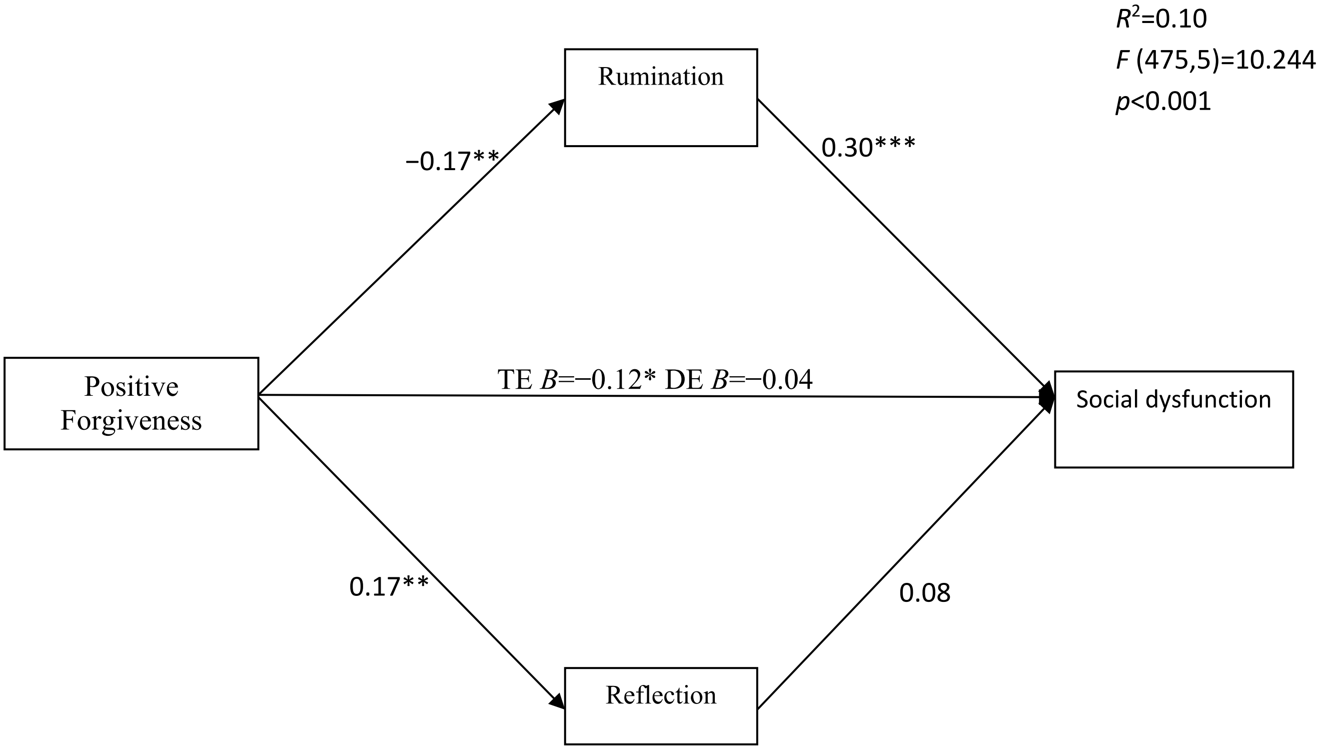

Figure S3. The indirect effect of positive forgiveness on social dysfunction via rumination and reflection .

*$p<0.05$; **$p<0.01$; ***$p<0.001$

IE (via Rumination and Reflection) – $B=-0.067$ $CI_{95\%}$[−0.104, −0.034]

IE (via Rumination) – $B=-0.052$ $CI_{95\%}$[−0.086, −0.025]

IE (via Reflection) – B=−0.015 $CI_{95\%}$[−0.037, −0.001]

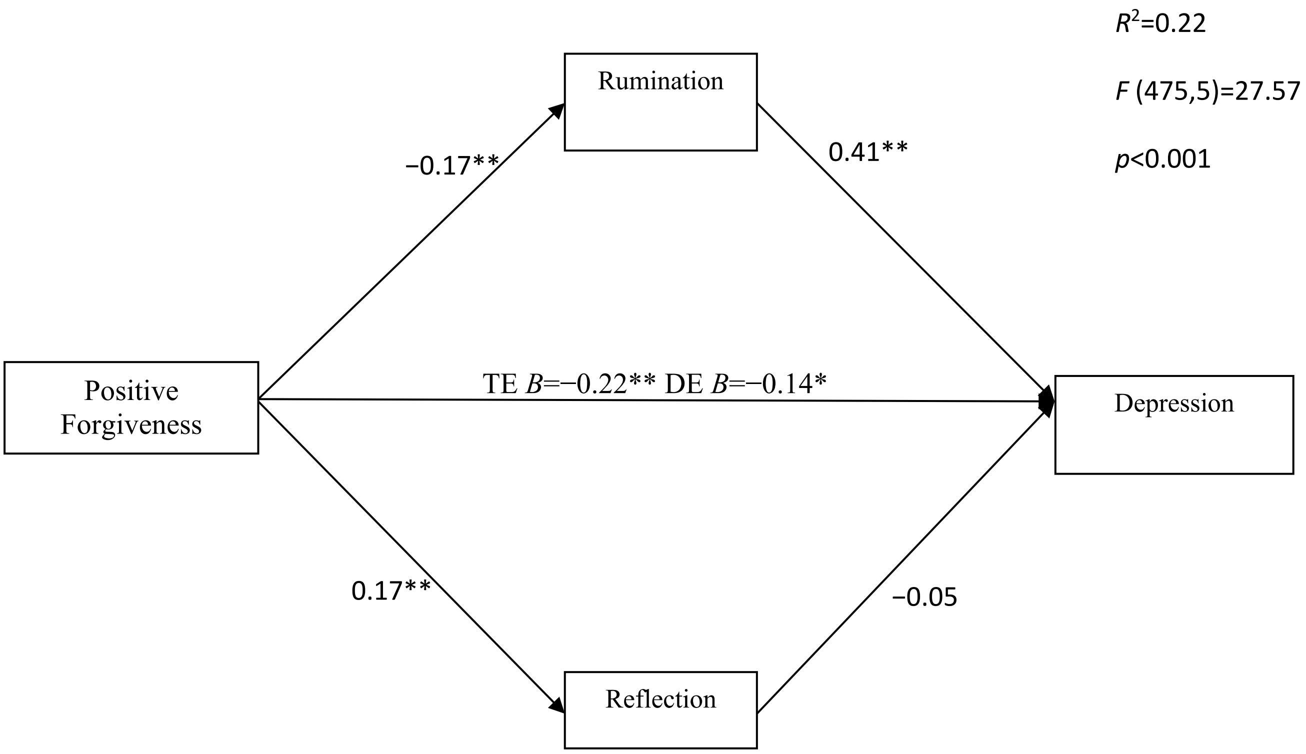

Figure S4. The indirect effect of positive forgiveness on depression via rumination and reflection .

*$p<0.05$; **$p<0.01$; ***$p<0.001$

IE (via Rumination and Reflection) – $B=-0.081$ $CI_{95\%}[-0.123, -0.040]$

IE (via Rumination) – $B=-0.071$ $CI_{95\%}[-0.110, -0.034]$

IE (via Reflection) – $B=-0.009$ $CI_{95\%}[-0.027, 0.004]$

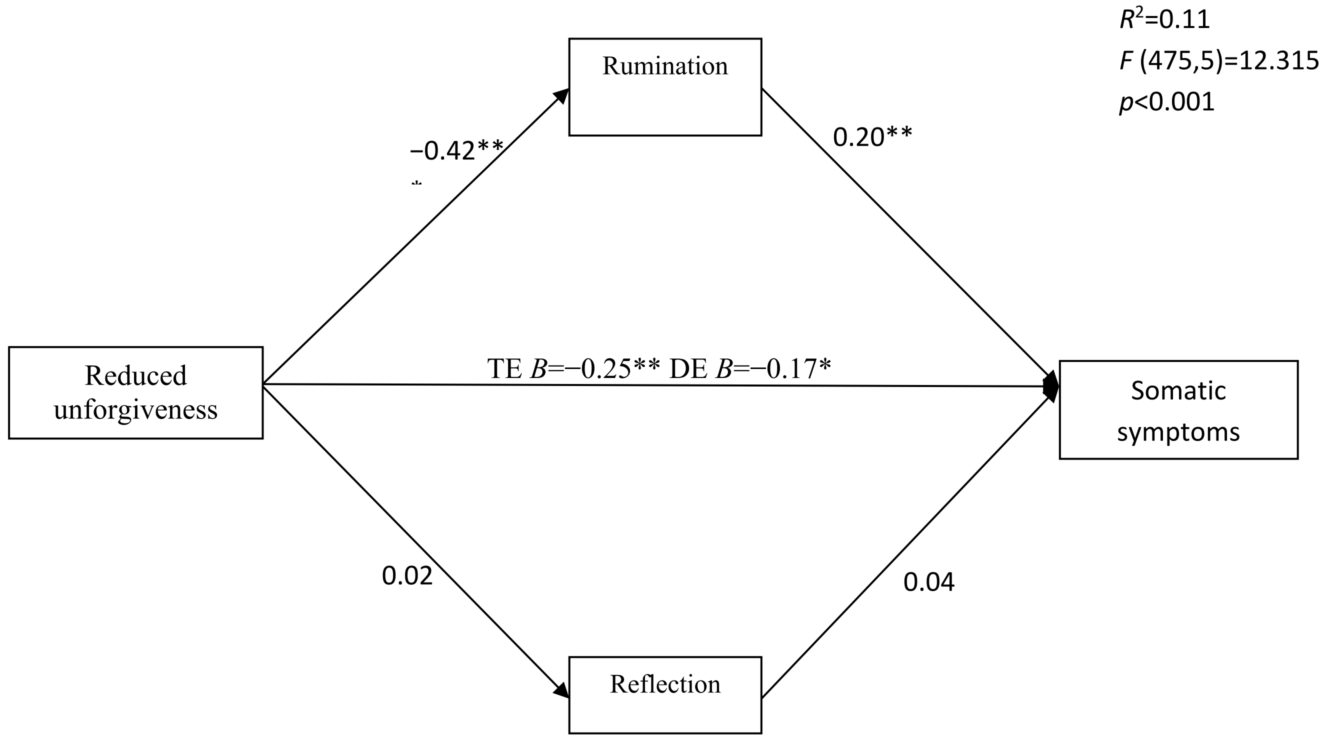

Figure S5. The indirect effect of reduced unforgiveness on somatic symptoms via rumination and reflection .

*p<0.05; **p<0.01; ***p<0.001

IE (via Rumination and Reflection) – B=−0.086 $CI_{95\%}$[−0.138, −0.037]

IE (via Rumination) – B=−0.086 $CI_{95\%}$[−0.136, −0.037]

IE (via Reflection) – B=−0.001 $CI_{95\%}$[−0.007, 0.005]

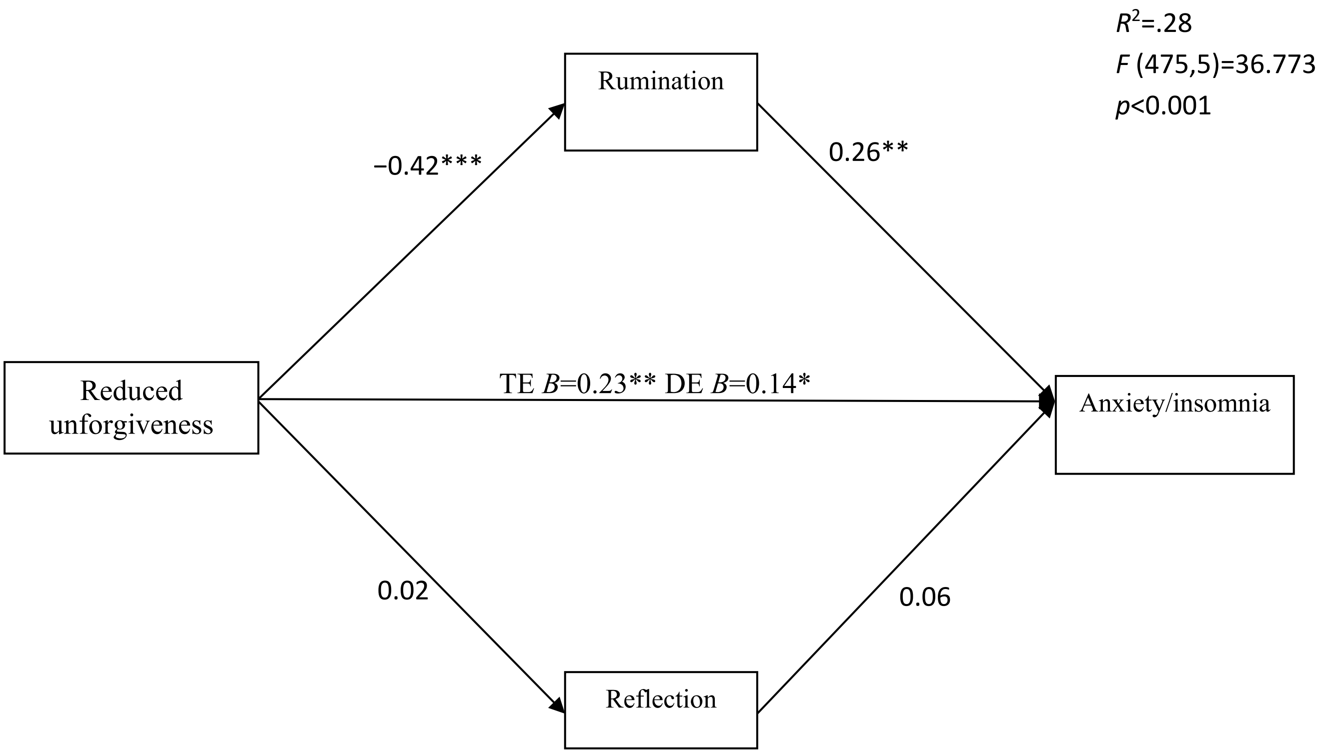

Figure S6. The indirect effect of reduced unforgiveness on anxiety/insomnia via rumination and reflection .

*p<0.05; **p<0.01; ***p<0.001

IE (via Rumination and Reflection) – B=−.058 $CI_{95\%}$[−.085, −.034]

IE (via Rumination) – B=−0.058 $CI_{95\%}$[−0.084, −0.034]

IE (via Reflection) – B=−0.001 $CI_{95\%}$[−0.004, 0.003]

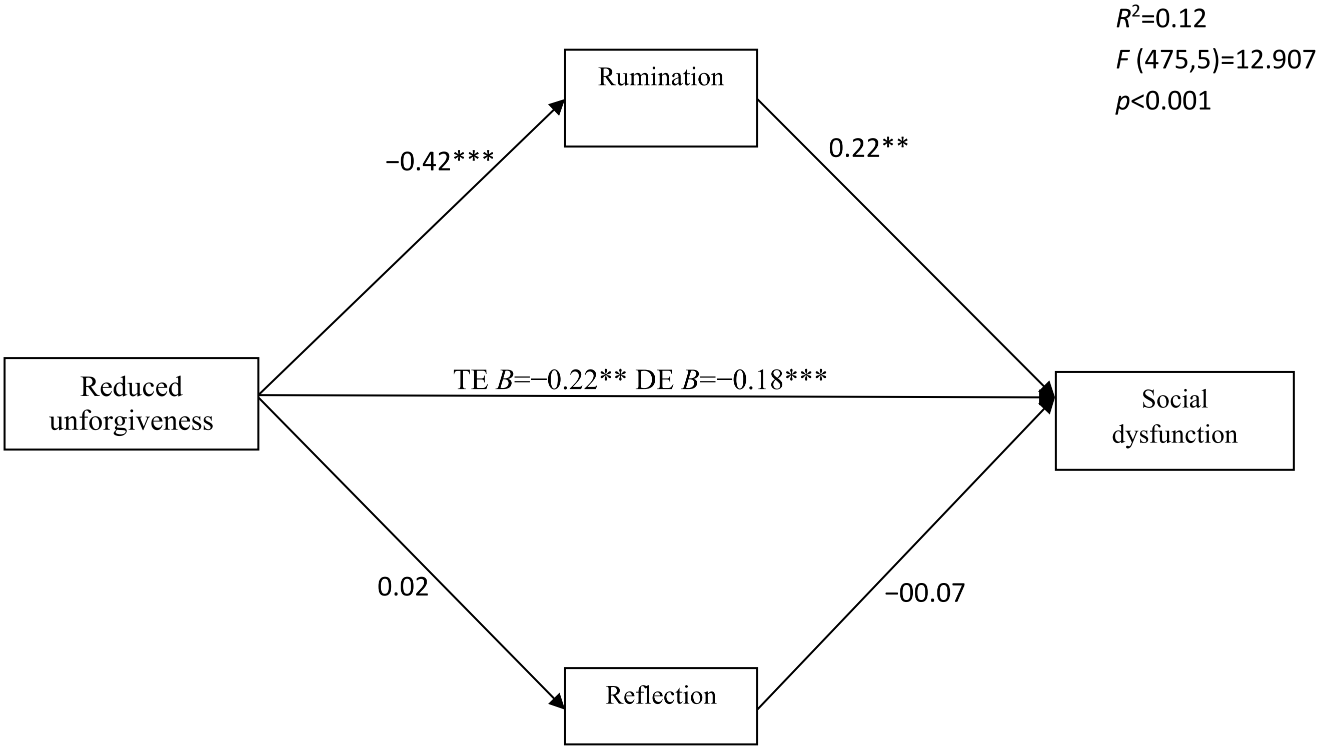

Figure S7. The indirect effect of reduced unforgiveness on social dysfunction via rumination and reflection.

*p<0.05; **p<0.01; ***p<0.001

IE (via Rumination and Reflection) – B=−0.096 $CI_{95\%}$[−0.146, −0.049]

IE (via Rumination) – B=−0.095 $CI_{95\%}$[−0.146, −0.048]

IE (via Reflection) – B=−0.001 $CI_{95\%}$[−0.009, 0.006]

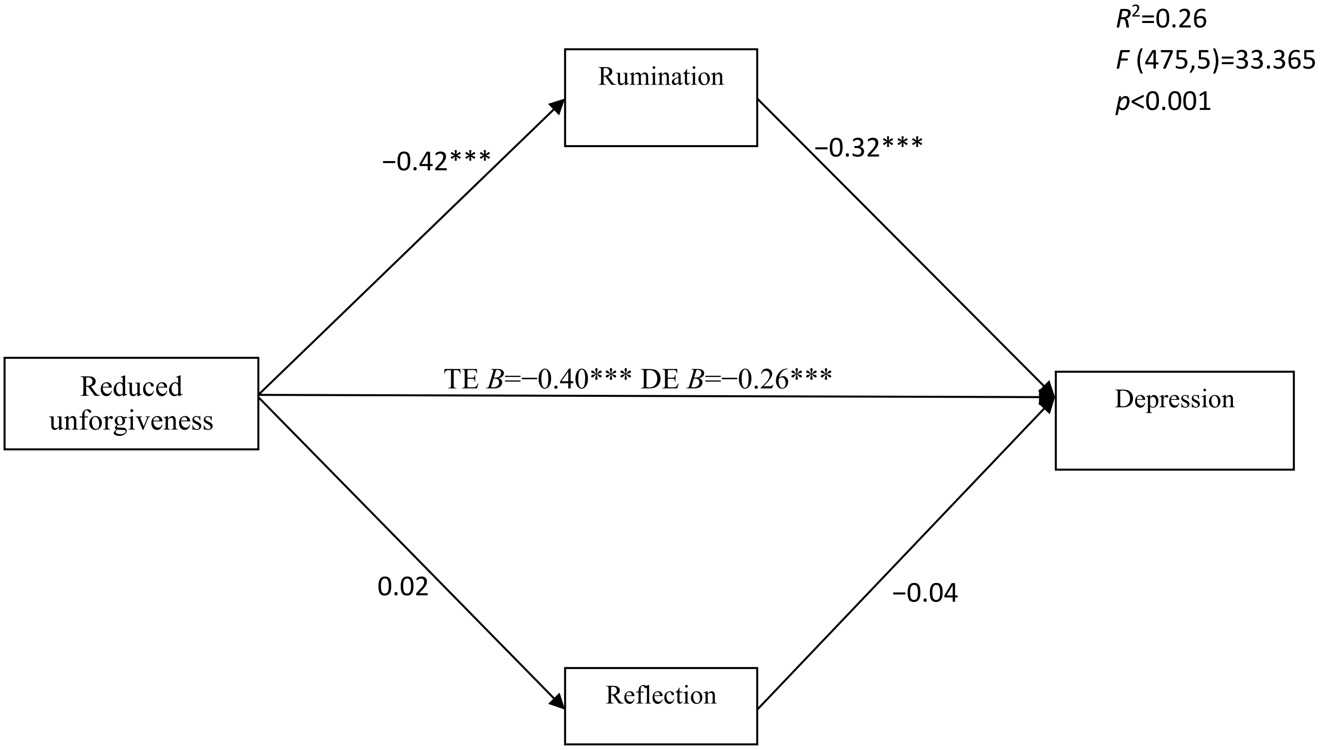

Figure S8. The indirect effect of reduced unforgiveness on depression via rumination and reflection.

*p<0.05; **p<0.01; ***p<0.001

IE (via Rumination and Reflection) – B=−0.135 $CI_{95\%}$[−0.181, −0.092]

IE (via Rumination) – B=−0.134 $CI_{95\%}$[−0.181, −0.092]

IE (via Reflection) – B=−0.001 $CI_{95\%}$[−0.007, 0.004]

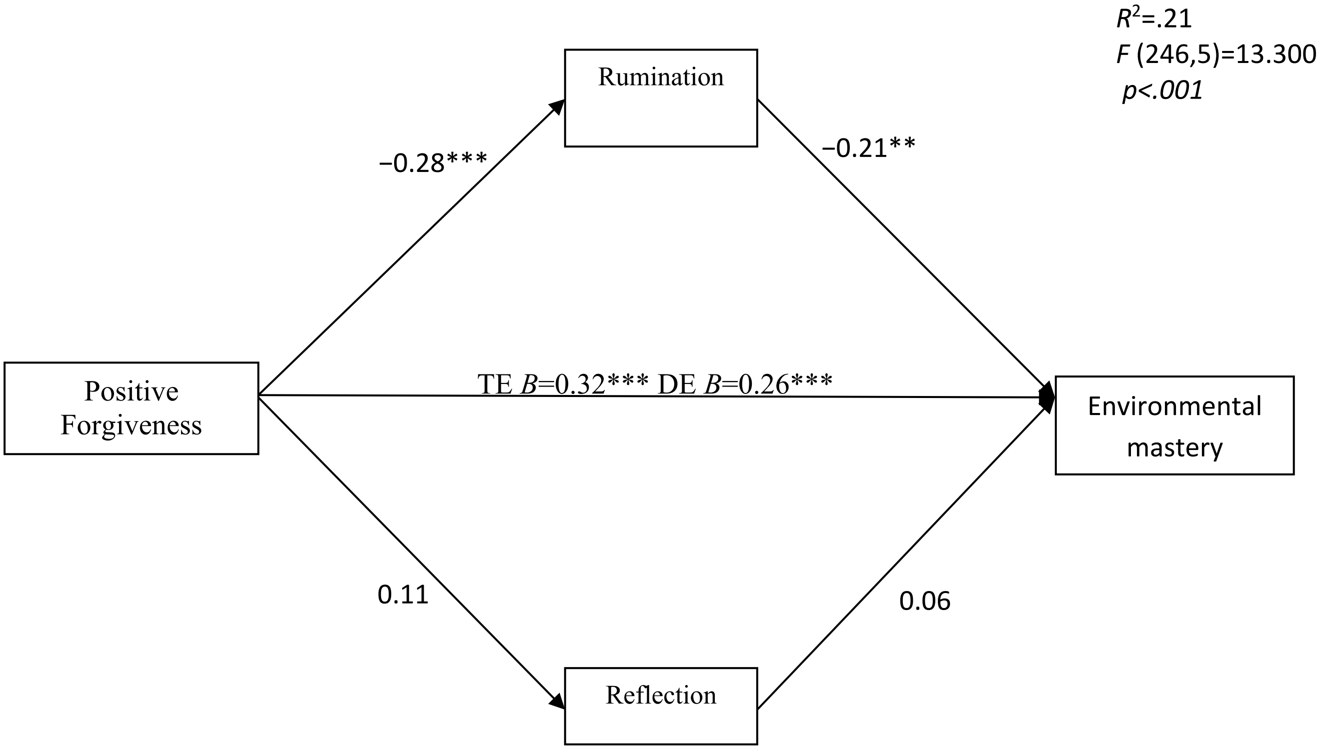

Figure S9. The indirect effect of positive forgiveness on environmental mastery rumination and reflection .

**p<0.01; ***p<0.001

IE (via Rumination and Reflection) – B=.065 $CI_{95\%}$[0.012, 0.125]

IE (via Rumination) – B=.025 $CI_{95\%}$[0.014, 0.115]

IE (via Reflection) – B=0.006 $CI_{95\%}$[−0.009, 0.026]

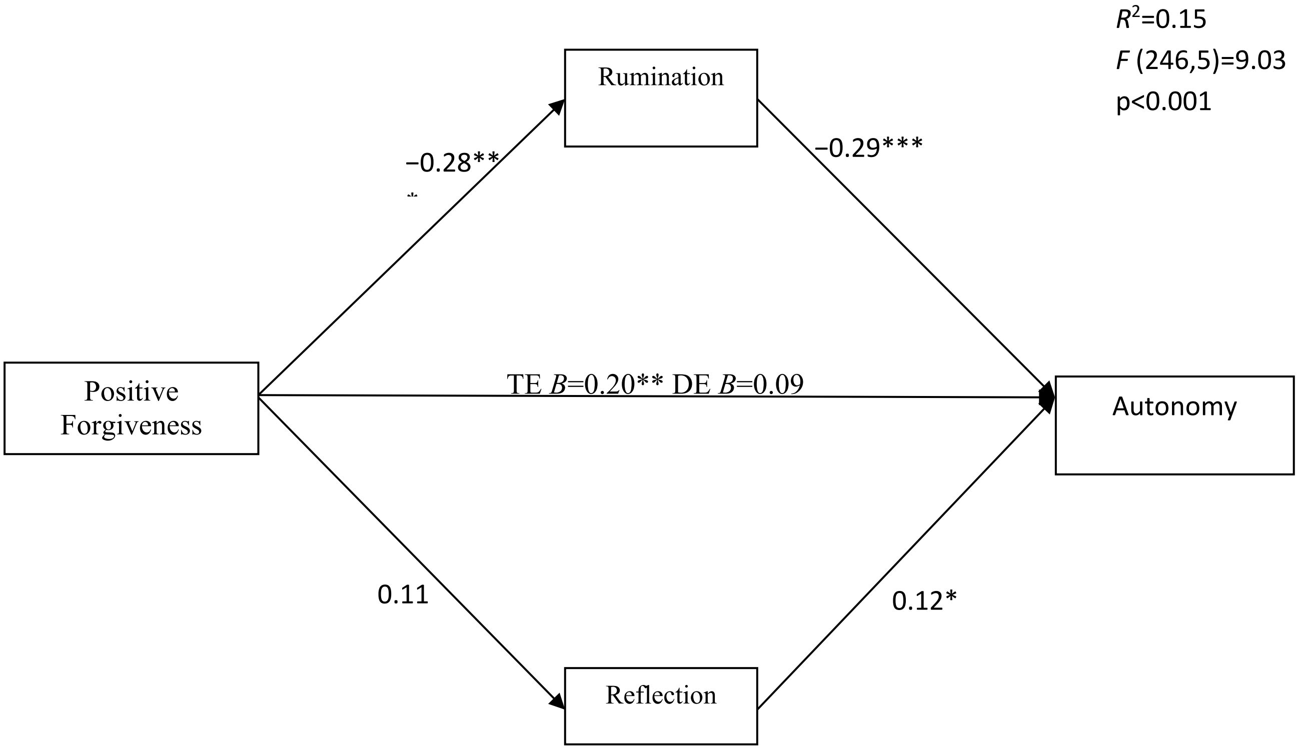

Figure S10. The indirect effect of positive forgiveness on autonomy via rumination and reflection.

**p*<.05; ***p*<.01; ****p*<.001

IE (via Rumination and Reflection) – *B*=0.096 $CI_{95\%}$[0.036, 0.171]

IE (via Rumination) – *B*=0.083 $CI_{95\%}$[0.030, 0.152]

IE (via Reflection) – *B*=0.013 $CI_{95\%}$[−0.006, 0.046]

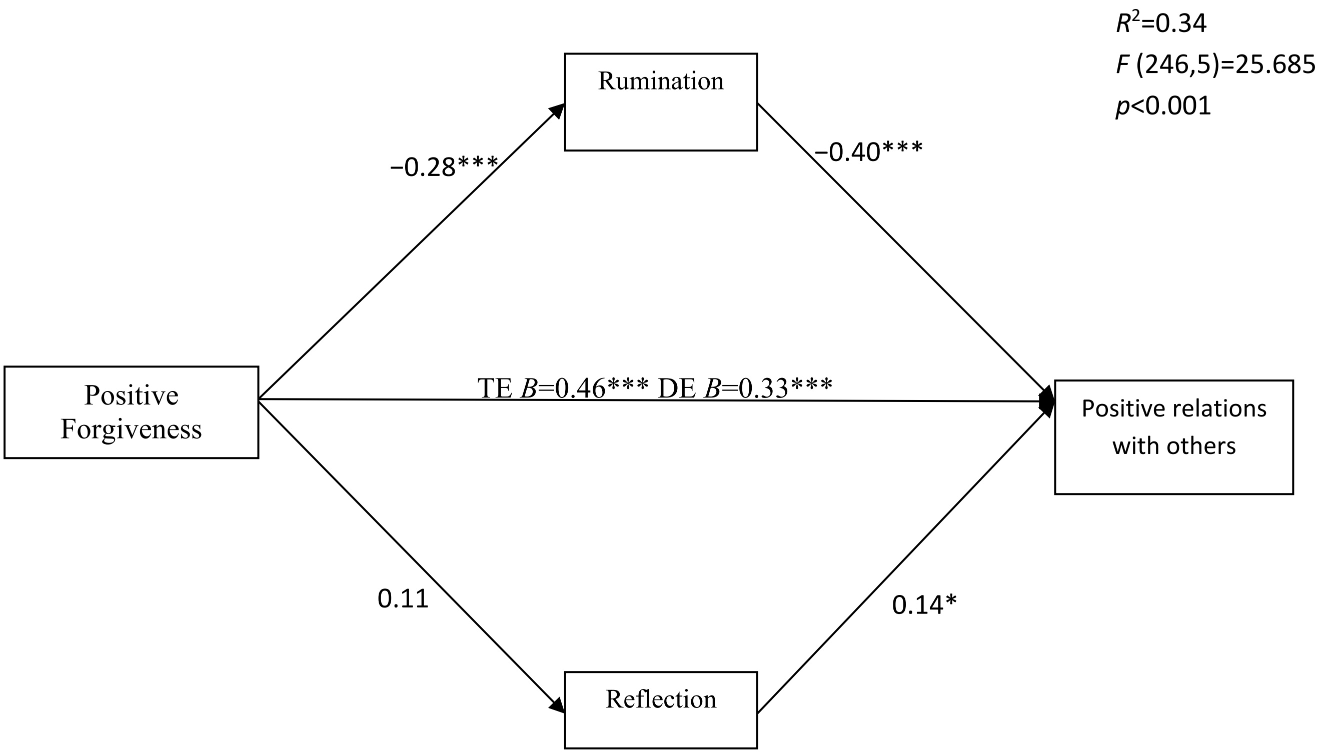

Figure S11. The indirect effect of positive forgiveness on positive relations with others via rumination and reflection .

*p<0.05; **p<0.01; ***p<0.001

IE (via Rumination and Reflection) – B=0.095 $CI_{95\%}$[0.039, 0.157]

IE (via Rumination) – B=0.091 $CI_{95\%}$[0.041, 0.146]

IE (via Reflection) – B=0.004 $CI_{95\%}$[−0.012, 0.024]

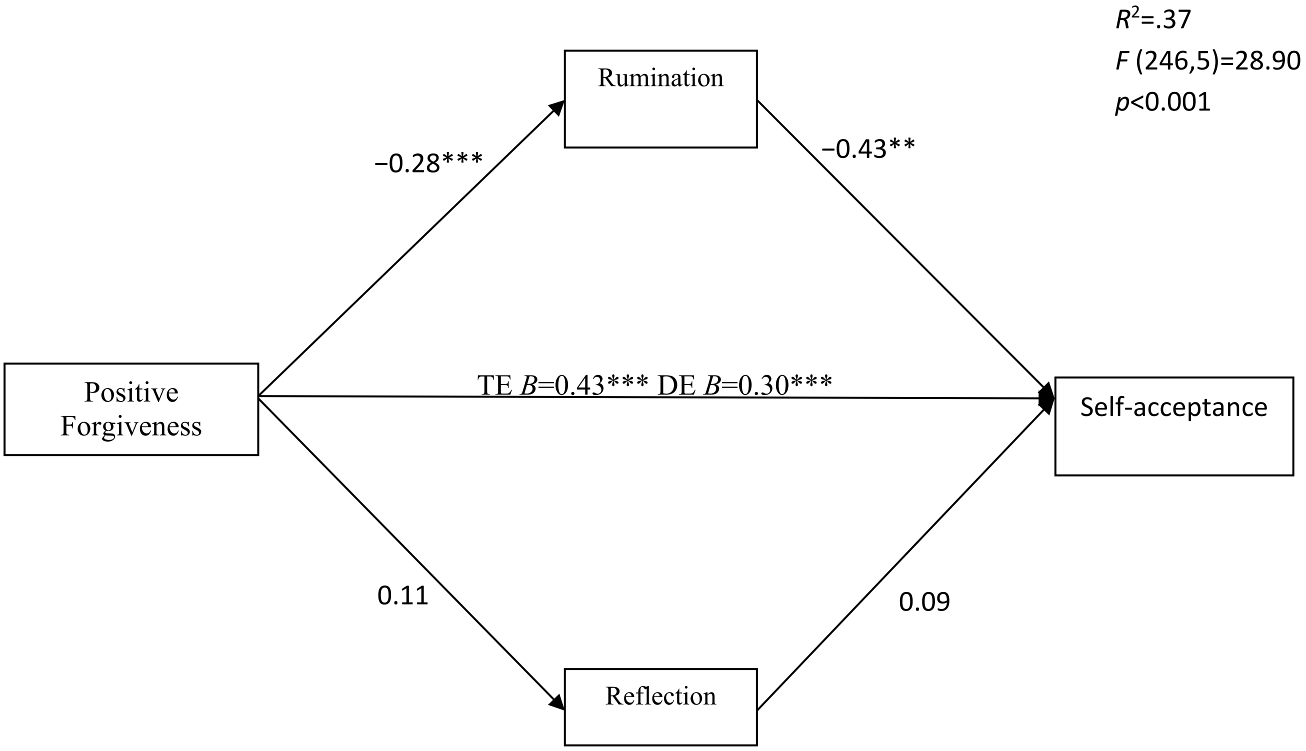

Figure S12. The indirect effect of positive forgiveness on self−acceptance via rumination and reflection.

*p<0.05; **p<0.01; ***p<0.001

IE (via Rumination and Reflection) – B=0.130 $CI_{95\%}$[0.071, 0.203]

IE (via Rumination) – B=0.123 $CI_{95\%}$[0.058, 0.192]

IE (via Reflection) – B=0.009 $CI_{95\%}$[−0.003, 0.034]

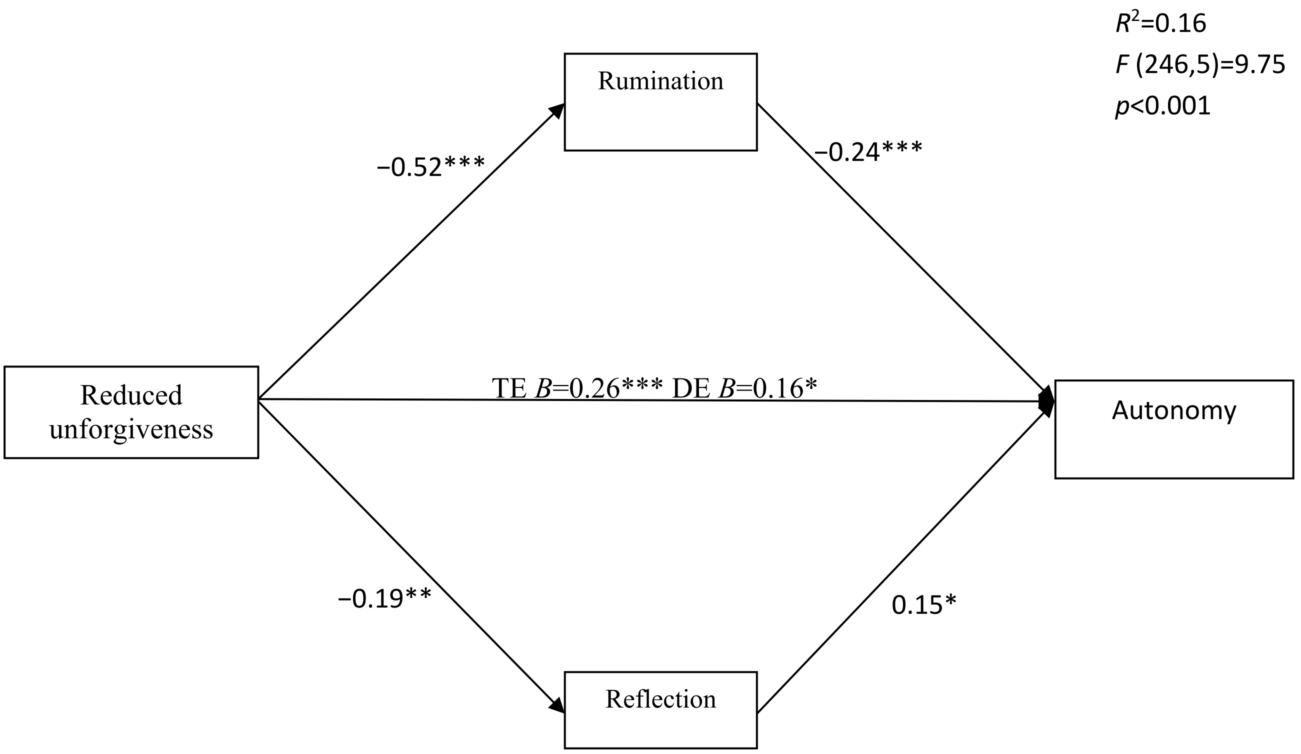

Figure S13. The indirect effect of reduced unforgiveness on autonomy via rumination and reflection.

*$p<0.05$; **$p<0.01$; ***$p<0.001$

IE (via Rumination and Reflection) – B=0.097 $CI_{95\%}$[0.013, 0.181]

IE (via Rumination) – B=0.127 $CI_{95\%}$[0.043, 0.212]

IE (via Reflection) – B=−0.029 $CI_{95\%}$[−0.073, 0.001]

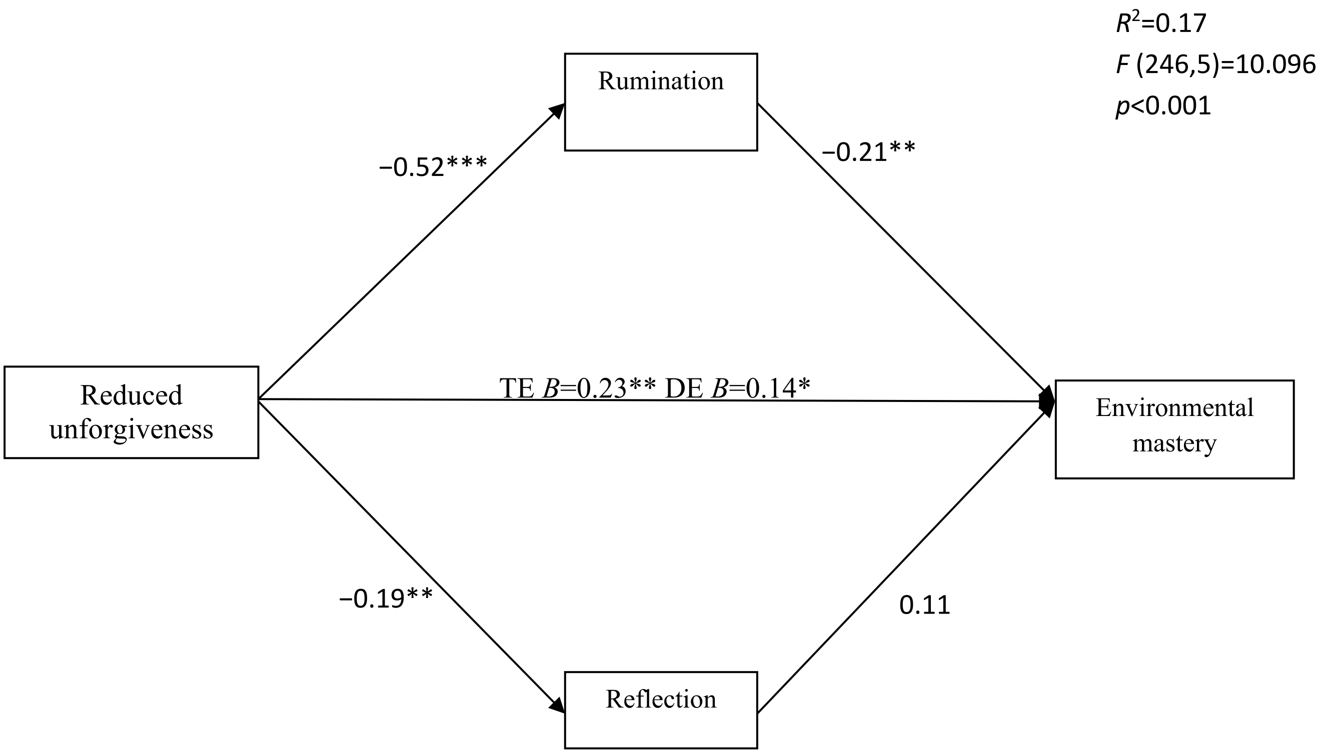

Figure S14. The indirect effect of reduced unforgiveness on environmental mastery via rumination and reflection.

*p<0.05; **p<0.01; ***p<0.001

IE (via Rumination and Reflection) – B=0.092 $CI_{95\%}$[0.001, 0.200]

IE (via Rumination) – B=0.114 $CI_{95\%}$[0.022, 0.227]

IE (via Reflection) – B=−0.022 $CI_{95\%}$[−0.054, 0.001]

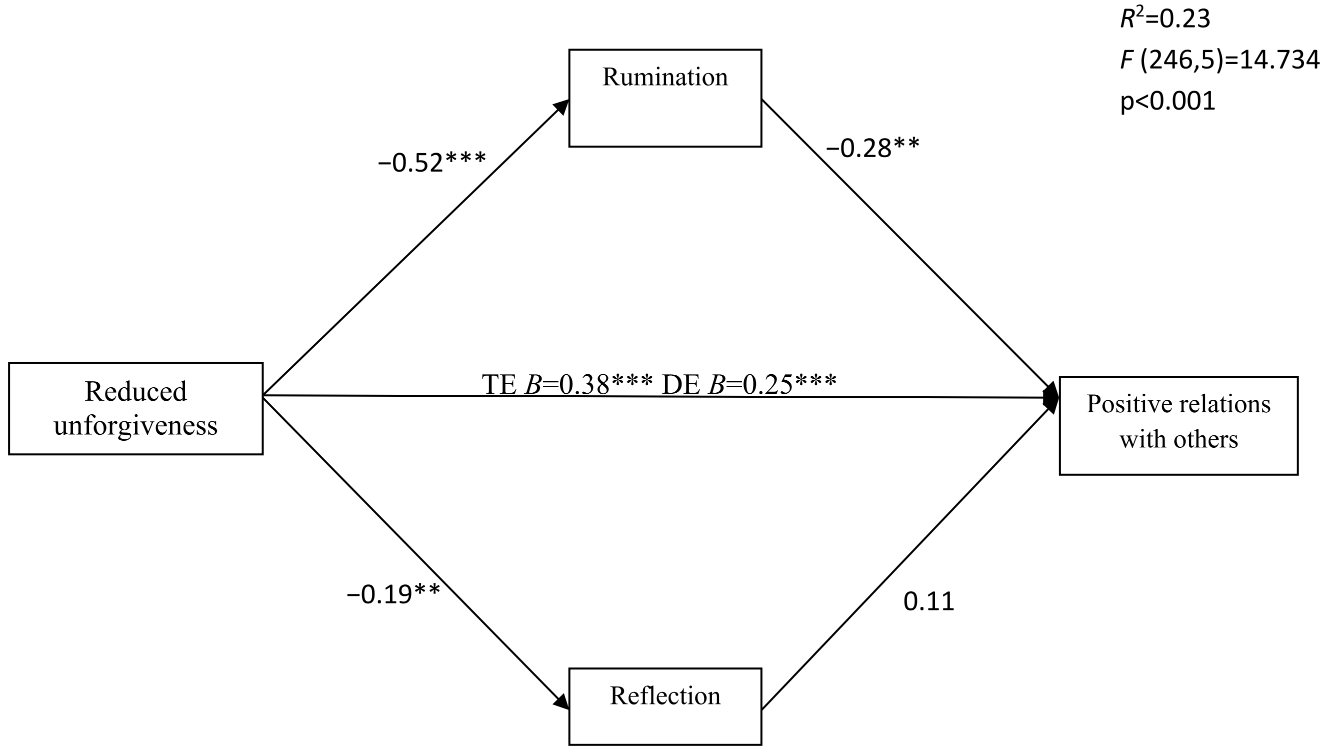

Figure S15. The indirect effect of reduced unforgiveness on positive relations with others via rumination and reflection.

$^*p<0.05$; $^{**}p<0.01$; $^{***}p<0.001$

IE (via Rumination and Reflection) – B=0.128 $CI_{95\%}$[0.054, 0.211]

IE (via Rumination) – B=0.150 $CI_{95\%}$[0.071, 0.237]

IE (via Reflection) – B=−0.021 $CI_{95\%}$[−0.049, 0.001]

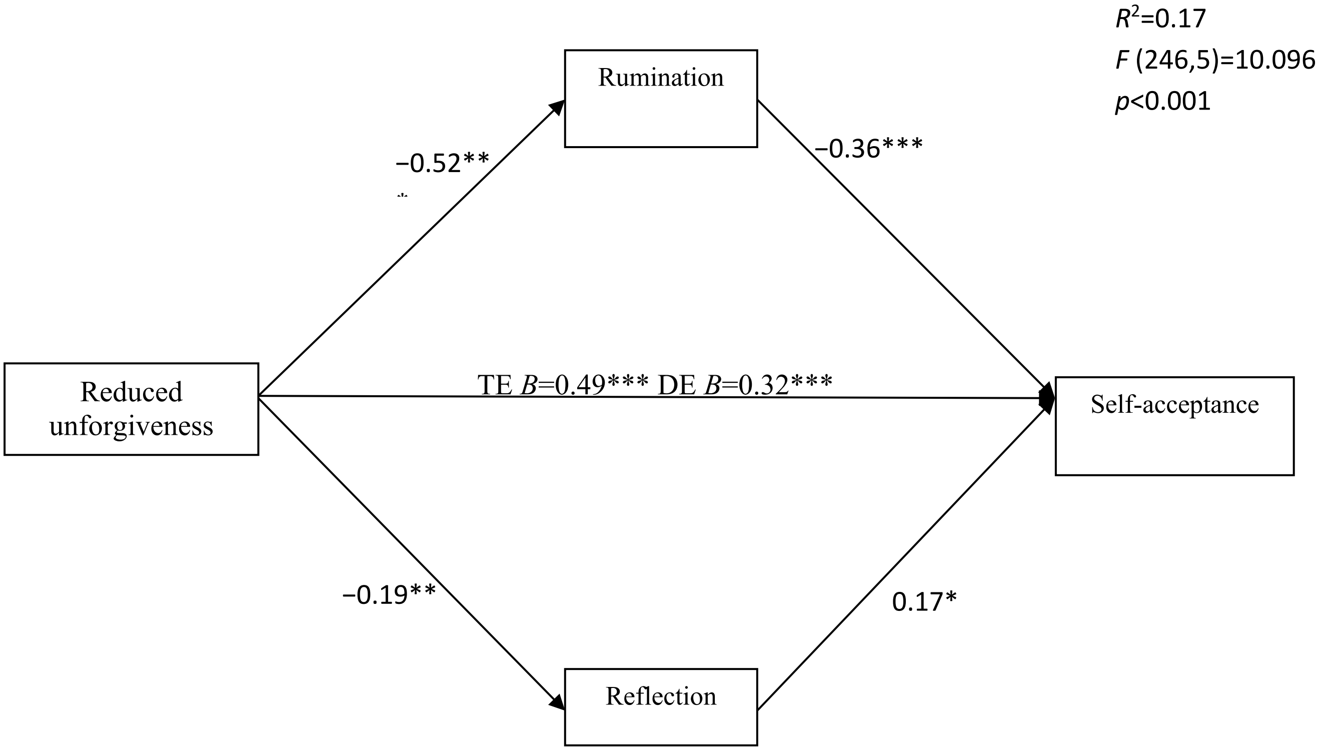

Figure S16. The indirect effect of reduced unforgiveness on self–acceptance via rumination and reflection.

**p*<0.05; ***p*<0.01; ****p*<0.001

IE (via Rumination and Reflection) – *B*=0.157 $CI_{95\%}$[0.083, 0.240]

IE (via Rumination) – *B*=0.190 $CI_{95\%}$[0.117, 0.274]

IE (via Reflection) – *B*=−0.032 $CI_{95\%}$[−0.075, −0.004]
